# Natalizumab-treated patients at high risk for PML persistently excrete JC polyomavirus

**DOI:** 10.1007/s13365-016-0449-0

**Published:** 2016-05-19

**Authors:** Milton H. Werner, DeRen Huang

**Affiliations:** 1 Inhibikase Therapeutics, Inc., 3350 Riverwood Pkwy SE, Ste 1900, Atlanta, GA 30339 USA; 2 Clinical Research, Neurology and Neuroscience Associates, Inc., 701 White Pond Dr., Akron, OH 44320 USA

**Keywords:** Progressive Multifocal Leukoencephalopathy (PML), JCV, Risk factors, Natalizumab, Tysabri

## Abstract

Sixty-three natalizumab-treated patients with relapsing multiple sclerosis were screened for JC polyomavirus (JCV) viruria. Urinary-positive patients were longitudinally sampled for up to 24 weeks. Using methods that distinguish encapsidated virus from naked viral DNA, 17.5 % of patients were found to excrete virus, consistent with the prevalence of urinary excretion in the general population. Unexpectedly, urinary excretion was predominantly seen (>73 %) in patients with high JC antibody index (≥2.0). Active JCV infection, therefore, tends to occur in natalizumab patients that carry a high risk factor for the development of disease, directly linking JC infection to the risk factors for PML development.

## Introduction

**Fig. 1:**
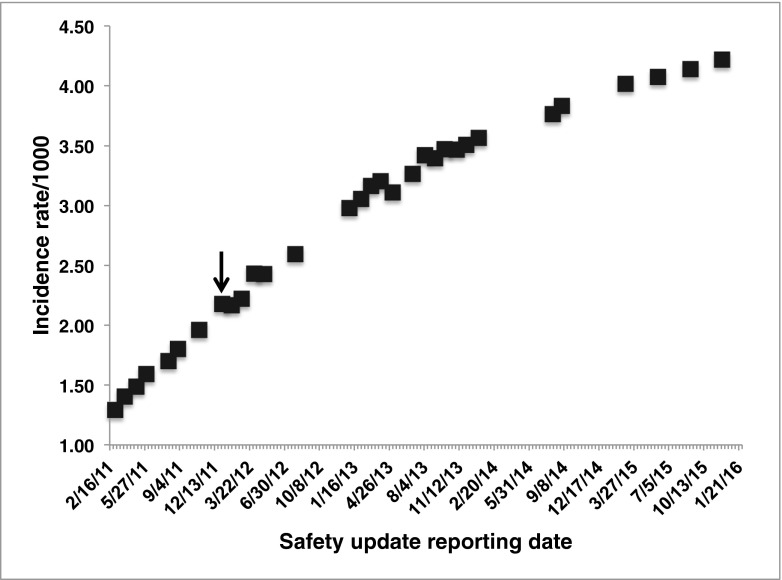
Rate of PML case occurrence and incidence during the era of JCV antibody testing in natalizumab-treated patients. Each *point* represents the overall incidence rate per 1000 natalizumab-treated patients at each safety update, computed from the total number PML cases and the reported total exposure to natalizumab in the post-market setting. There has been a modest reduction in incidence rate in the last 24 months. The approval, in January, 2012, for JC antibody titer/index test is indicated with the *arrow*

Natalizumab is a highly efficacious therapy for patients with relapsing multiple sclerosis (MS) but is associated with induction of Progressive Multifocal Leukoencephalopathy (PML), a debilitating or fatal brain infection of the JC polyomavirus (JCV) (Wollebo et al. 2015). The risk for development of PML is correlated with a patient’s prior exposure to the virus (Rudick et al. 2010; Bloomgren et al. 2012) quantified from anti-JCV antibodies and expressed as the antibody index (AI) (Plavina et al. 2014). Ninety-six percent of PML cases in controlled studies arose in patients with AI > 0.9, and 84 % of cases arose in patients with AI > 1.5 (Plavina et al. 2014). Utilization of the anti-JCV antibody titer/index in treatment algorithms would therefore be expected to result in a reduced rate of PML. However, in the nearly 4 years since antibody titer or AI has been used as a means of risk stratification, there has been only a modest reduction in the incidence of PML arising from natalizumab treatment (Fig. 1) (Biogen Idec and Biogen Natalizumab Safety Updates March 4, 2011 through September 4, 2015; www.biogen.com).

A possible deficiency in the AI risk stratification approach is its dissociation from JCV infection itself. Indeed, antibodies are merely markers of the history of immune responses to JCV-specific antigen exposure, which could reflect prior exposure to virus from weeks to many years in the past. Assessment of concurrent viral infection might be more accurate in determining the real-time viral status in patients. The accessible compartment in living patients from which JC viral load can be routinely measured is the genitourinary compartment and the bone marrow (Ryschkewitsch et al. 2004). While the pathogenic form of JC virus in the CNS may originate from virus in the genitourinary compartment, the genomic rearrangements associated with the brain-infective form are rarely seen in urine (Van Loy et al. 2013) and it is often reported that urinary excretion of JCV is intermittent (Lanzillo et al. 2014; Delbue et al. 2015; Domínguez-Mozo et al. 2013; Berger et al. 2013). We interrogated the urine of 63 natalizumab-treated patients to evaluate whether viruria was intermittent, whether virus, or only viral DNA, was found and to evaluate the relationship between urinary virus and known risk factors for progression to PML. We found that MS patients treated with natalizumab who excreted JC virus did so consistently, that the bulk of the viral DNA signal is likely to derive from encapsidated virus and that the majority of patients with persistent excretion are just those patients with high AI. Thus, the majority of patients in the high-risk category for progression to PML also have an active JCV infection.

## Patients and methods

The study was approved by an institutional review board (Quorum IRB, Seattle, WA), and informed consent was obtained for each patient by his/her treating physician prior to providing medical information or screening sample. Study participants were natalizumab-treated MS patients at least 6 months on therapy and screened for the presence or absence of JCV viruria. Patients with a positive screening test were given 24 weekly urine collection kits, and first-morning samples were collected 7 ± 2 days apart. AI, gender, number of natalizumab-infusions, and prior or concurrent treatments for MS or other co-morbid indications were recorded. Weekly urine samples were self-collected and shipped at ambient temperature to Inhibikase Therapeutics (Atlanta, GA and Cambridge, MA).

Sample processing was performed to discriminate virus-associated viral DNA from all other forms in the sample. For total viral DNA, 1 mL unfiltered, uncentrifuged urine was heated to 60 °C for 10 min in 2 mM dithiothreitol (DTT), 50 mM Na_2_EDTA, and 50 mM K_2_EGTA. These conditions disrupt encapsidated virus in phosphate-buffered saline (Neu et al. 2010). For virus-associated viral DNA, 1 mL unfiltered, uncentrifuged urine was treated with 25 u/mL Benzonase® (EMD Millipore), a DNA exo-/endonuclease, for 10 min at 37 °C, followed by heat/DTT/EDTA/EGTA treatment as for processing of total viral DNA. Cell-associated viral DNA that was not encapsidated was determined to be absent or making a minimal contribution to the observed qPCR signal in these samples since the viral DNA in urine resisted Benzonase® treatment even following overnight incubation in 25 % (*v/v*) non-protein-denaturing detergents (e.g., M-Per, ThermoFisher), a condition wherein Benzonase® remained active. Total viral DNA and virus-associated viral DNA were measured from duplicate processed samples each week by quantitative polymerase chain reaction (qPCR) at a reference laboratory, Viracor IBT. Viracor’s proprietary PCR protocol primes within a highly conserved region of the large T-antigen using proprietary primer sequences closely related to previously published methods (Ryschkewitsch et al. 2004). Viracor’s limit of quantitation (LOQ), defined as a day-to-day qPCR variation of ≤30 %, is 151 genome equivalents/mL urine (Geq/mL). The qPCR amplification protocol is proprietary to Viracor IBT, but fails to amplify non-target DNA samples, even from other human polyomaviruses like BK, in validation experiments. Details of the amplification and validation criteria may be requested directly from Viracor IBT. Any sample measuring below 151 was recorded as having a signal <151 and could be distinguished from samples that had no signal at all.

## Results

Sixty-three natalizumab-treated patients were screened for the presence of JCV viruria. Eleven patients screened positive (17.5 %) and provided up to 24 weekly urine samples processed as described. Longitudinal sampling revealed that urinary viral load varied between 2.2 and 7.8 log_10_ Geq/mL (Table 1) among the screen-positive patients, but within a patient, viral load measured consistently (Table 1). Only one patient, 9027, displayed intermittent excretion, with virus-associated viral DNA found in 18 of 24 samples and close to the LOQ (Table 1). Three screen-negative patients with AI of 1.8, 1.5, or 1.35 also provided 24 weekly samples. Two of these screen-negative patients had a total viral DNA signal in 1 of 24 samples; the third patient displayed a total viral DNA signal in 2 of 24 samples. However, the total viral DNA signal degraded following Benzonase® treatment, indicating that these samples did not contain encapsidated virus. Thus, with one sample’s exception out of 72 collected, screen-negative patients remained negative throughout the study period.

**Table 1:** Longitudinal sampling of urinary virus in natalizumab patients

Patient ID	AI	No. of infusions	Log_10_ total viral DNA ± S.D. (no. of samples)^a^	Log_10_ virus-associated viral DNA ± S.D. (no. samples)^a^
8002	0.65	18	5.51 ± 0.45 (24)	5.16 ± 0.61 (24)
9027	0.98	19	3.13 ± 0.44 (24)	3.27 ± 0.5 (24)
8010	1.7	22	5.98 ± 0.41 (24)	6.00 ± 0.55 (24)
8006	2.0	8	7.03 ± 0.44 (24)	6.93 ± 0.66 (24)
9040	2.1	12	4.08 ± 0.61 (24)	4.01 ± 0.76 (24)
8013	2.4	51	7.83 ± 0.19 (24)	7.84 ± 0.20 (24)
8001	2.8	38	5.91 ± 0.46 (24)	5.80 ± 0.53 (24)
8005	2.6	51	4.02 ± 0.68 (24)	3.97 ± 0.53 (24)
9033	3.1	13	6.22 ± 0.58 (24)	6.12 ± 0.58 (24)
9028	3.3	6	6.91 ± 0.46 (24)	6.71 ± 0.81 (24)
8003	3.8	101	6.80 ± 0.59 (24)	6.77 ± 0.62 (24)

Total or virus-associated viral DNA is defined in “Patients and methods”. AI shown was that last measured prior to the screening sample having been collected. The number of infusions represents the total number of natalizumab infusions prior to the screening sample

^a^The number of weeks of collection completed is indicated in parentheses

**Fig. 2:**
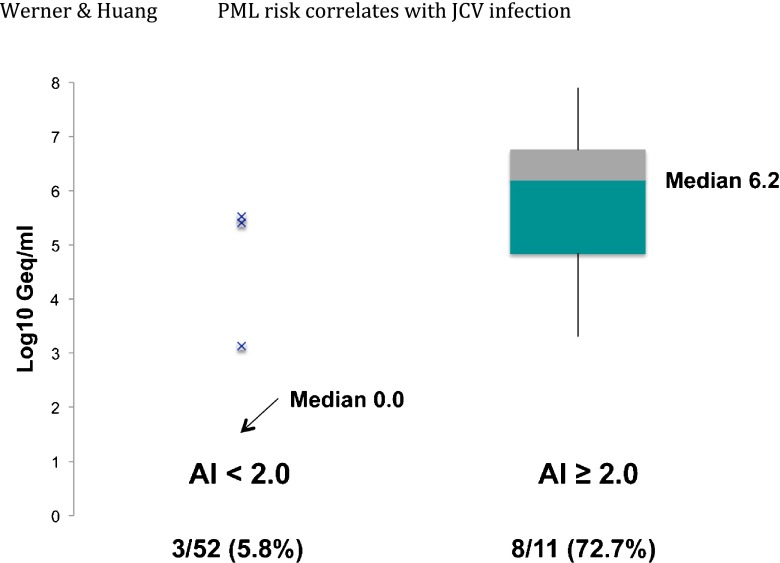
Correlation of antibody index with JC infection in 63 natalizumab-treated patients. Box-whisper plots correlate patients with high antibody index to urinary excretion of JC virus. The median level of excretion for patients with AI ≥ 2.0 is log_10_ = 6.2 (interface of *green and gray* boxes representing the second and third quartiles of the population of viral excretors). Only 3 of 52 patients (5.8 %) with AI < 2.0 displayed evidence of a persistent JCV infection in the urine. The correlation of AI ≥ 2.0 with urinary excretion is statistically significant (*t* test, *P* < 0.001). No statistically significant correlation is observed between the number of infusions and the observed viral load

Urine processing is likely reducing the viral DNA signal in patient samples to just the virus-associated viral DNA, allowing for a direct measure of patient viral load shed from the genitourinary compartment. Correlates were sought between urinary viral load and PML risk factors. Three of 52 patients with AI < 2.0 were viruric (5.8 %), in contrast to the 72.7 % of patients with AI ≥ 2.0 that consistently excreted virus (Fig. 2). Since 84 % of PML cases in controlled studies arose in patients with AI > 1.5, the majority of patients with active JC infection detected in the urine were just those patients considered to be in the high-risk category for progression to PML (Plavina et al. 2014; McGuigan et al. 2015).

## Discussion

Among the three established risk factors for progression to PML during natalizumab treatment (AI, treatment duration, prior immunosuppressant use) (Plavina et al. 2014; McGuigan et al. 2015), AI has been used to determine which patients should initiate natalizumab therapy and at what point a patient should be transitioned to alternatives. Given that PML cases have continued to increase with only a modest reduction since antibody titer/AI measurements were introduced (Fig. 1) (www.biogen.com), augmenting risk algorithms with additional risk factors seems imperative. The prevalence of JCV viruria among natalizumab-treated patients is reported to cover a wide range (18–87 %) (Delbue et al. 2015; Lanzillo et al. 2014; Laroni et al. 2012; Bellizzi et al. 2013; Rinaldi et al. 2010), but no association between viruria prevalence and progression to PML has been found. The strong correlation demonstrated herein between AI and JCV viruria indicates that natalizumab-treated patients with active JCV infection commonly fall into a high-risk category for progression to clinical disease (Plavina et al. 2014). Ferrante and co-workers made a similar observation, finding that more than 80 % of patients with viruria were also seropositive for JCV-specific antibodies, but could not assign these observations to low or high PML risk groups since the AI had not yet been introduced into clinical practice (Delbue et al. 2015). For this reason, measurement of urinary excretion has not accompanied analysis of AI in PML cases either. It had been previously concluded that viruria is not correlated with serostatus or antibody titer in PML cases (Rudick et al. 2010) and not even the prevalence of JCV excretion in PML cases has been systematically measured. The clear correlation between AI and viruria reported herein should force re-examination of this issue in PML cases.

The introduction of new handling procedures for patient urine has resulted in consistent measurement of patient viral loads for genitourinary virus. These methods may overcome the reported observation that JCV viruria is intermittent (see Clausi et al. 2015; Delbue et al. 2015; Lanzillo et al. 2014; Domínguez-Mozo et al. 2013; Saundh et al. 2010 for examples). The introduction of DNAse and chemical treatments suggests that actual, encapsidated virus can now be quantified in patient samples. Intermittent excretion made the measure of urinary JCV of little diagnostic benefit, leaving it largely ignored in the approach to mitigating PML risk. Given that appropriate interrogation of the urine reveals a consistent signal that likely represents encapsidated virus, that the viral load is high and strongly correlated with high antibody titer, and the fact that the level of viruria increases with progression to PML (Delbue et al. 2015; Bellizzi et al. 2013; Domínguez-Mozo et al. 2015) suggest urinary JCV has clinical value. Active JCV infection in the urine can often be detected prior to JCV antibodies (Laroni et al. 2012; Lanzillo et al. 2014), directing attention to those patients that may already be heading down the path to neurotropic JCV. The conundrum has always been where the transformation from genitourinary (i.e., archetype) to neurotropic JCV takes place. Correlation between the site(s) of viral replication, the prevalence of viral excretion, the level of viral replication, and the antibody titer/index, as described herein, may point the finger at the primary JCV reservoir as the site of origin for neurotropic JCV, but whose independence from the reservoir is established after initial escape (Reid et al. 2011; Van Loy et al. 2015). Consistent with this hypothesis, genomic rearrangements and VP1 capsid mutations, both implicated in enabling virus to migrate into other tissues and organs, have only been identified from plasma or serum, PBMC subsets, and PML brain or cerebrospinal fluid (CSF) (Reid et al. 2011; Gorelik et al. 2011). PML remains rare, a consequence of the rarity of events necessary to enable JCV mobilization and migration during natalizumab treatment. The selective pressure to mutate will increase for the virus once it leaves its traditional replicative milieu, driving the types of transformations that have now been documented in both coding and non-coding regions of the viral genome. But therapeutic intervention may best be focused at the origin of these events and along the path leading to the CNS. With the ability to directly measure patient viral load from an accessible compartment now established, a biomarker exists from which the response to antiviral therapy can begin to be measured in natalizumab patients.
